# Supplementary material for: LaGrACE: estimating gene program dysregulation with latent regulatory network
Source: Mol Syst Biol. 2025 Jun 30;21(9):1263–81. doi: 10.1038/s44320-025-00115-3 (PMC12405484; doi:10.1038/s44320-025-00115-3)
Supplement: Supplementary file 1 — Appendix [file 44320_2025_115_MOESM1_ESM.pdf]

Appendix for

LaGrACE: Estimating gene program dysregulation with latent regulatory network

Minxue Jia[1,2], Haiyi Mao[1,2], Mengli Zhou[1,4], Yu-Chih Chen[1,2,3], Panayiotis V. Benos[1,2,4, *]

[1]Department of Computational and Systems Biology, University of Pittsburgh School of Medicine, Pittsburgh, Pennsylvania, USA; [2]Joint CMU-Pitt PhD Program in Computational Biology, Pittsburgh, Pennsylvania, USA; [3]UPMC Hillman Cancer Center, Pittsburgh, Pennsylvania, USA; [4]University of Florida, Gainesville, Florida, USA

Table of Contents

Appendix Text 3

Appendix Text S1. Calculation of purity scores. 3

Appendix Table 4

Appendix Table S1. Clinical characteristics of breast cancer patients vary across clusters. 4

Appendix Table S2. Nottingham histologic grade (NHG) was stratified by clusters on SCAN-B datasets 5

Appendix Table S3. Discrimination indexes of cox proportional hazard model on survival and recurrent events 6

Appendix Table S4. cluster-specific survival factors 7

Appendix Table S5. Runtime of LaGrACE. 8

Appendix Figure 9

Appendix Figure S1. LaGrACE captures dose-response signal from BMS-345541 treatment at single-cell resolution. 9

Appendix Figure S2. LaGrACE captures dose-response signal from nutlin-3a treatment at single-cell resolution. 10

Appendix Figure S3. LaGrACE captures dose-response signal from vorinostat treatment at single-cell resolution. 11

Appendix Figure S4. Breast Cancer subject survival analysis and recurrent event analysis for clusters with similar molecular subtype composition on two breast cancer datasets. 12

Appendix Figure S5. Clinical variables among clusters identified on METABRIC dataset. 13

Appendix Figure S6. Separation of METABRIC breast cancer samples and Kaplan-Meier estimate of survival and recurrent event by ssGSEA's and Pathifier's clusters 14

Appendix Figure S7. Biological process and molecular mechanism difference among identified clusters in breast cancer. 15

Appendix Figure S8. Heatmap of scaled predicted cell type fractions inferred using CIBERSORTx 16

Appendix Figure S9. The cluster specific causal graphs for luminal clusters (1, 2 & 3). 17

Appendix Figure S10. FOXM1 inhibitors inhibit the cell migration of breast cancer cells. (Scale bar: 500 μm) 19

Appendix Figure S11. Association between LaGrACE cluster and clinical variables. 19

Appendix Figure S12. Association between LaGrACE cluster and white blood cell type fraction. 20

Appendix Figure S13: Kaplan-Meier survival curve analysis for COPD patients. 21

Appendix Figure S14: COPD module score and LEF1 regulon score in CD4 and CD8 T cells from human lung tissue. 22

Appendix Text

Appendix Text S1. Calculation of purity scores.

We adapted the concept of "purity score" from the cell type purity metric described in [1]. Purity is defined as the percentage of cells within a neighborhood that share the same label (e.g., a specific cell type).

1. **Constructing the Neighborhoods:**
   We begin by creating a k-nearest neighbor (KNN) graph based on high-dimensional features (such as gene expression, gene program scores, or LaGrACE divergence scores). Each cell is represented as a node, and edges connect each cell to its k-nearest neighbors, determined using principal component analysis (PCA) on the chosen input features.
2. **Defining Neighborhood Purity:**
   Each neighborhood is defined as the set of cells directly connected to a given "index cell" in the KNN graph. To calculate the purity for a given neighborhood, we:
   - Identify the most abundant label within that neighborhood.
   - Compute the purity score as the fraction of cells in the neighborhood that share this majority label.
3. **Applying to Our Dataset:**
   Instead of cell type labels, we use treatment-related labels (e.g., treatment duration or drug concentration) as our "cell type" labels. By doing so, we assess whether cells exposed to the same treatment conditions cluster together and exhibit similar gene expression or gene program patterns. This allows us to measure how well a given representation (e.g., raw gene expression, gene programs, or LaGrACE features) preserves treatment-related differences.

We use the *Milo* package [1] to implement this approach. Milo provides functions to construct KNN graphs, build neighbourhoods, and compute purity scores on single-cell datasets.

References:

1. Dann, E., Henderson, N.C., Teichmann, S.A., Morgan, M.D., & Marioni, J.C. (2022). Differential abundance testing on single-cell data using k-nearest neighbor graphs. *Nature Biotechnology*, 40, 245–253.

Appendix Table

Appendix Table S1. Clinical characteristics of breast cancer patients vary across clusters. P-values were calculated with a Kruskal-Wallis test or Wilcoxon rank-sum test for continuous and ordinal variables, or a Chi-squared test for discrete and binary variables.

	Clinical Feature	All Clusters	Cluster 2 vs 3	Cluster 4 vs 5
**METABRIC**	**Nottingham histologic index**	**<2.2e-16**	**0.0031**	**0.048**
	**Tumor Cellularity**	**2.64E-12**	**0.01732**	**0.01605**
	**Inferred Menopausal State**	**6.86E-15**	**0.0002635**	**0.00964**
**SCAN-B**	**Nottingham histologic grade**	**<2.2e-16**	**<2.2e-16**	
	**KI67 status**	**<2.2e-16**	**<2.2e-16**	

Appendix Table S2. Nottingham histologic grade (NHG) was stratified by clusters on SCAN-B datasets. G2 is equivalent to 6~7 Nottingham histologic index (NPI), and G3 is equivalent to 8~9 NPI.

NHG	G2	G3
0	336	369
1	768	2
2	498	9
3	122	75
4	2	8

Appendix Table S3. Discrimination indexes of cox proportional hazard model on survival and recurrent events

Input	Task	C-index	CPE
Molecular Subtype	METABRIC Survival	0.628 (se = 0.013)	0.606 (se = 0.011)
**LaGrACE**		**0.642 (se = 0.013)**	**0.630 (se = 0.012)**
ssGSEA		0.629 (se = 0.013)	0.612 (se = 0.012)
Pathifier		0.606 (se = 0.014)	0.587 (se = 0.013)
Molecular Subtype	METABRIC Distant Relapse	0.618 (se = 0.014)	0.600 (se = 0.012)
**LaGrACE**		**0.654 (se = 0.013)**	**0.634 (se = 0.012)**
ssGSEA		0.630 (se = 0.013)	0.612 (se = 0.013)
Pathifier		0.604 (se = 0.014)	0.585 (se = 0.013)
Molecular Subtype	METABRIC Local Relapse	0.614 (se = 0.021)	0.583 (se = 0.018)
**LaGrACE**		**0.626 (se = 0.019)**	**0.597 (se = 0.020)**
ssGSEA		0.622 (se = 0.021)	0.589 (se = 0.020)
Pathifier		0.616 (se = 0.021)	0.592 (se = 0.020)
Molecular Subtype	SCANB Survival	0.561 (se = 0.020)	0.557 (se = 0.016)
**LaGrACE**		**0.617 (se = 0.019)**	**0.622 (se = 0.018)**

Appendix Table S4. cluster-specific survival factors

Gene Program	Cluster	Hazard Ratio	P value
Galactose Metabolism	**1**	**0.58**	**4.70E-04**
	2	0.76	0.14
	3	0.99	0.97
Integrin-Linked Kinase (ILK) Signaling	1	1.09	0.66
	**2**	**1.27**	**7.77E-04**
	3	1.16	0.12
E2F Targets-2	1	1.13	0.5
	2	1.16	0.2
	3	0.77	**0.022**
Regulation of RhoA Activity	1	1.07	0.67
	2	1.2	0.1
	**3**	**1.68**	**4.44E-05**

Appendix Table S5. Runtime of LaGrACE. Two datasets were tested using a 3 GHz 6-Core Intel Core i5 iMac: scRNA-seq data from 5,530 A549 cells treated with SAHA (including 1,005 reference samples) with 3,000 highly variable genes, and simulated high-dimensional data with 4,000 samples (1,000 reference samples) containing 2,500 continuous and 25 discrete variables.

Step	scRNA-seq Data (seconds)	Simulated Data (seconds)
Step 1	60.8	69.2
Step 2	5.5	5.4
Step 3 & 4	1.5	1.8

Appendix Figure

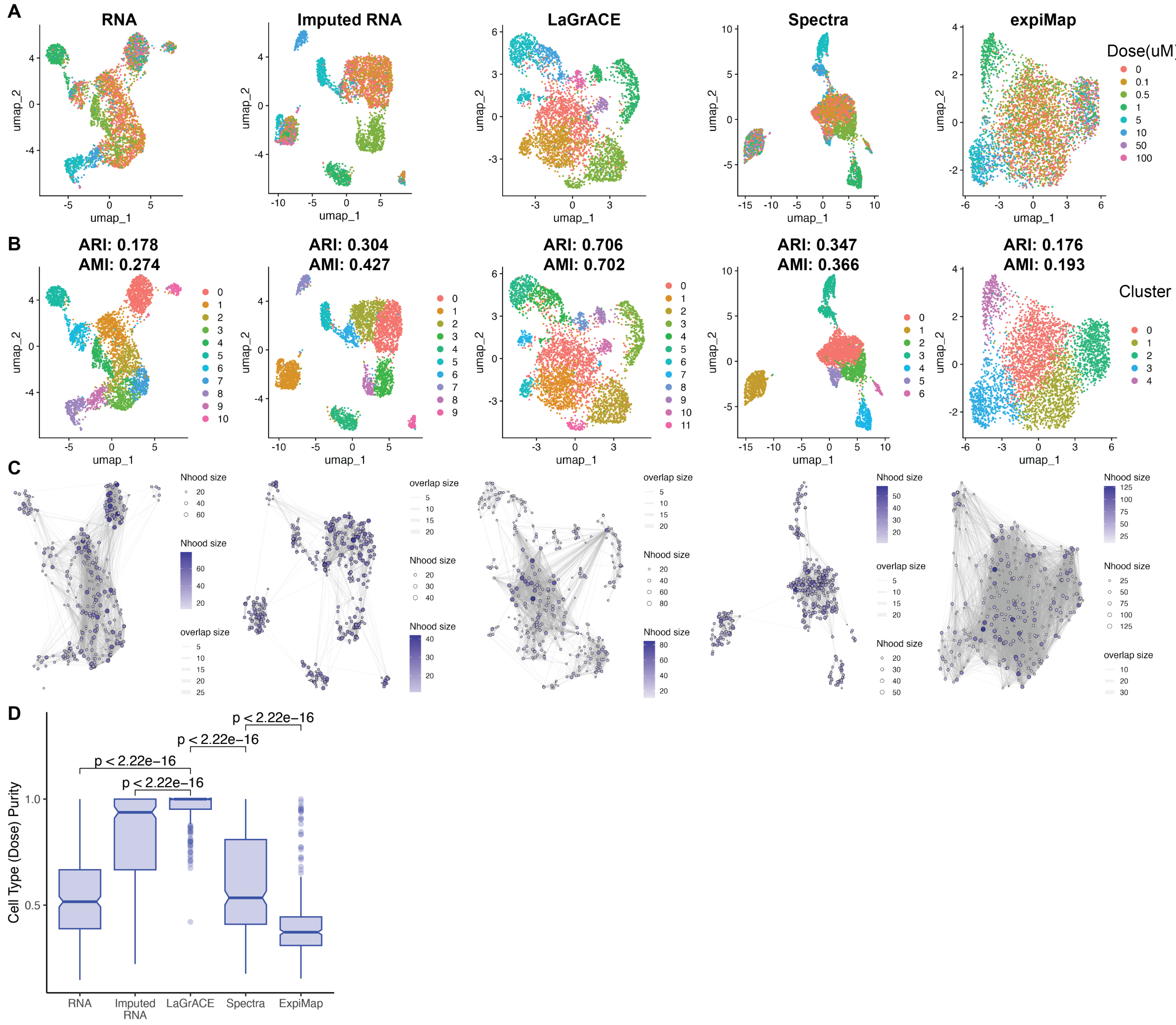

Appendix Figure S1. LaGrACE captures dose-response signal from BMS-345541 treatment at single-cell resolution. A549 lung adenocarcinoma cells were treated with BMS345541 (an inhibitor of nuclear factor κB–dependent transcription) for 24 hours.

(A) A549 cells visualized and colored by dose on UMAP embeddings computed based on unimputed RNA profiles, RNA profiles imputed by SCVI, LaGrACE features and gene set scores inferred by Spectra and ExpiMAP.

(B) UMAP plot of A549 cells colored by clusters based on unimputed RNA (ARI: 0.178, AMI: 0. 274), imputed RNA (ARI: 0.304, AMI: 0.427), LaGrACE features (ARI: 0.706, AMI: 0.702), Spectra gene set scores (ARI: 0.347, AMI: 0.366), and ExpiMAP gene set scores (ARI: 0.176, AMI: 0.193).

(C) Neighborhood graph constructed based on unimputed RNA profiles, RNA profiles imputed by SCVI, LaGrACE features and gene set scores inferred by Spectra and ExpiMAP using Milo.

(D) Boxplot of cell type purity score for single cell Neighborhoods constructed using Milo.

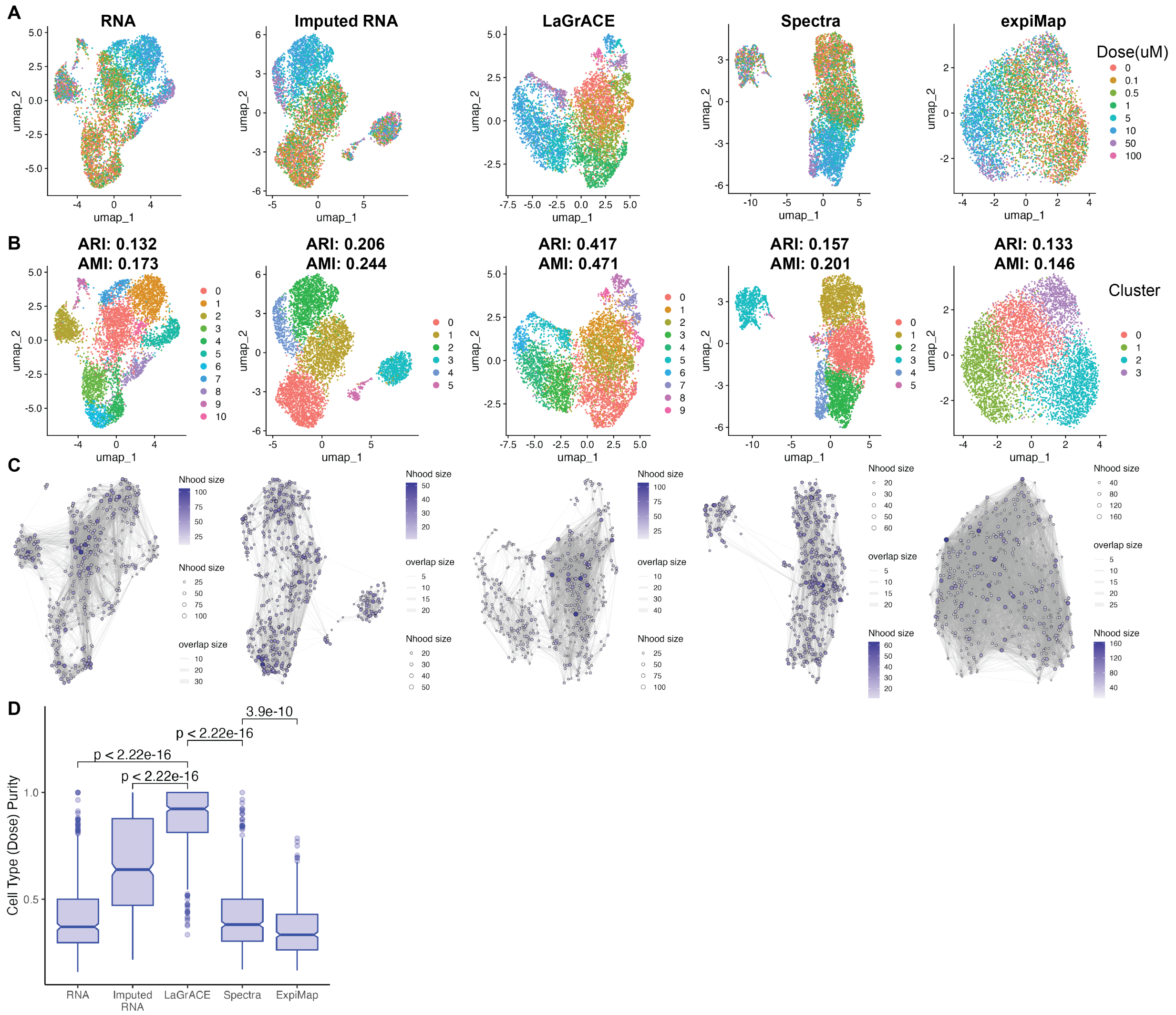

Appendix Figure S2. LaGrACE captures dose-response signal from nutlin-3a treatment at single-cell resolution. A549 lung adenocarcinoma cells were treated with nutlin-3a (a p53-Mdm2 antagonist) for 24 hours.

(A) A549 cells visualized and colored by dose on UMAP embeddings computed based on unimputed RNA profiles, RNA profiles imputed by SCVI, LaGrACE features and gene set scores inferred by Spectra and ExpiMAP.

(B) UMAP plot of A549 cells colored by clusters based on unimputed RNA (ARI: 0.132, AMI: 0.173), imputed RNA (ARI: 0.206, AMI: 0.244), LaGrACE features (ARI: 0.417, AMI: 0.471), Spectra gene set scores (ARI: 0.157, AMI: 0.201), and ExpiMAP gene set scores (ARI: 0.133, AMI: 0.146).

(C) Neighborhood graph constructed based on unimputed RNA profiles, RNA profiles imputed by SCVI, LaGrACE features and gene set scores inferred by Spectra and ExpiMAP using Milo.

(D) Boxplot of cell type purity score for single cell Neighborhoods constructed using Milo.

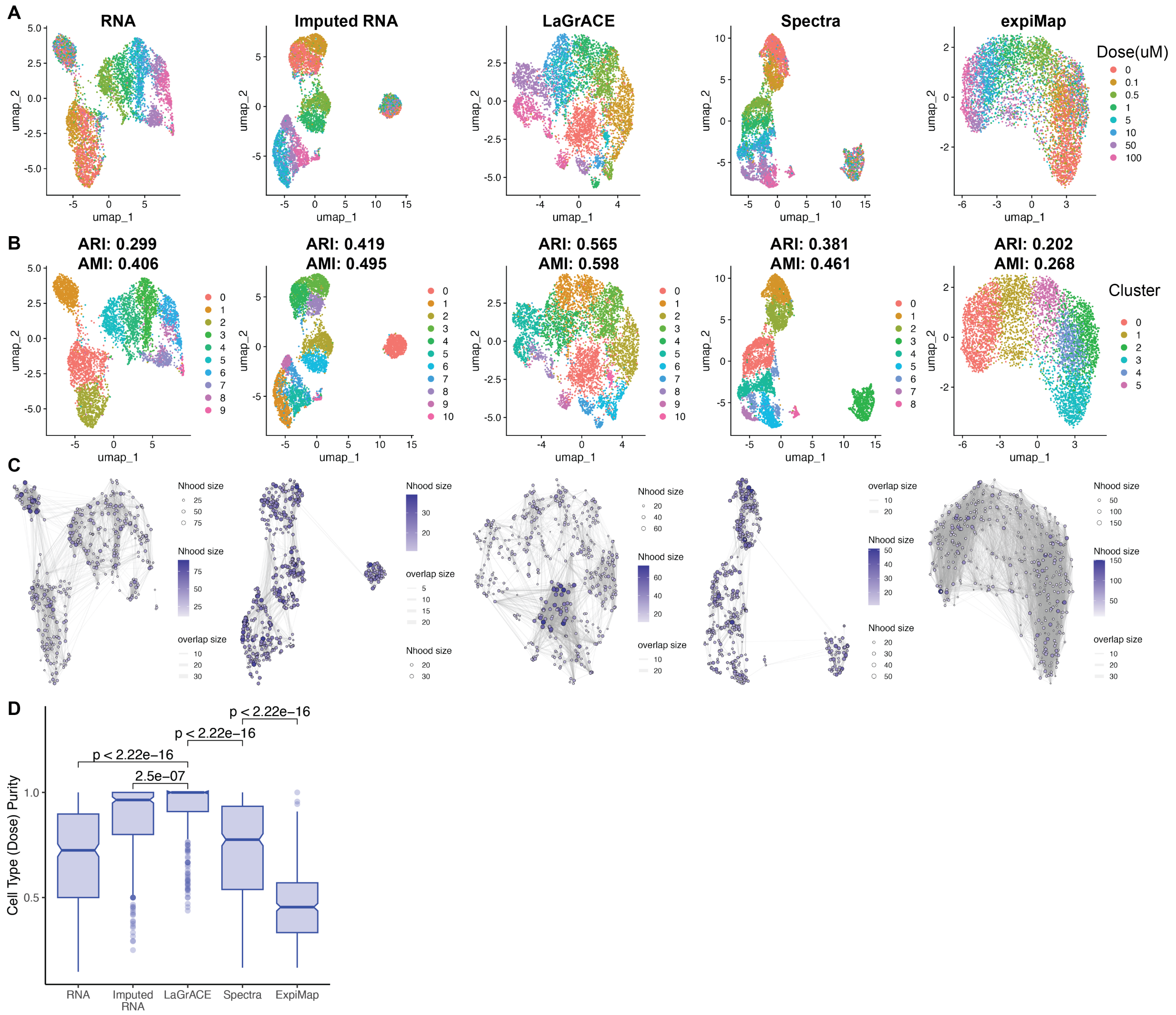

Appendix Figure S3. LaGrACE captures dose-response signal from vorinostat treatment at single-cell resolution. A549 lung adenocarcinoma cells were treated with suberoylanilide hydroxamic acid (SAHA), an HDAC inhibitor, for 24 hours.

(A) A549 cells visualized and colored by dose on UMAP embeddings computed based on unimputed RNA profiles, RNA profiles imputed by SCVI, LaGrACE features and gene set scores inferred by Spectra and ExpiMAP.

(B) UMAP plot of A549 cells colored by clusters based on unimputed RNA (ARI: 0.299, AMI: 0.406), imputed RNA (ARI: 0.419, AMI: 0.495), LaGrACE features (ARI: 0.565, AMI: 0.598) , Spectra gene set scores (ARI: 0.381, AMI: 0.461), and ExpiMAP gene set scores (ARI: 0.202, AMI: 0.268).

(C) Neighborhood graph constructed based on unimputed RNA profiles, RNA profiles imputed by SCVI, LaGrACE features and gene set scores inferred by Spectra and ExpiMAP using Milo.

(D) Boxplot of cell type purity score for single cell Neighborhoods constructed using Milo.

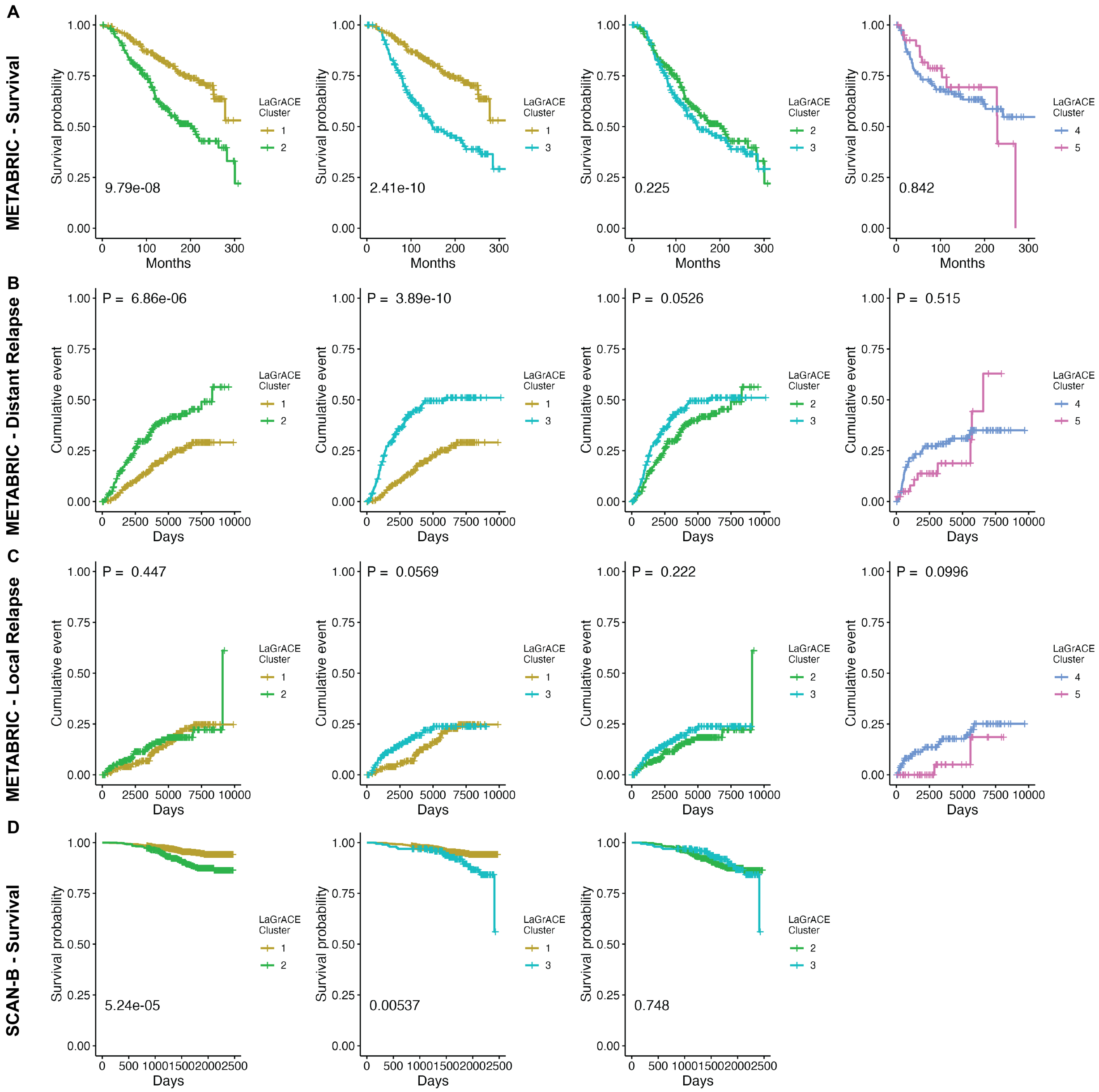

Appendix Figure S4. Breast Cancer subject survival analysis and recurrent event analysis for clusters with similar molecular subtype composition on two breast cancer datasets. Luminal samples were grouped into cluster 1, 2 & 3; Claudin-low samples were grouped into clusters 4 & 5.

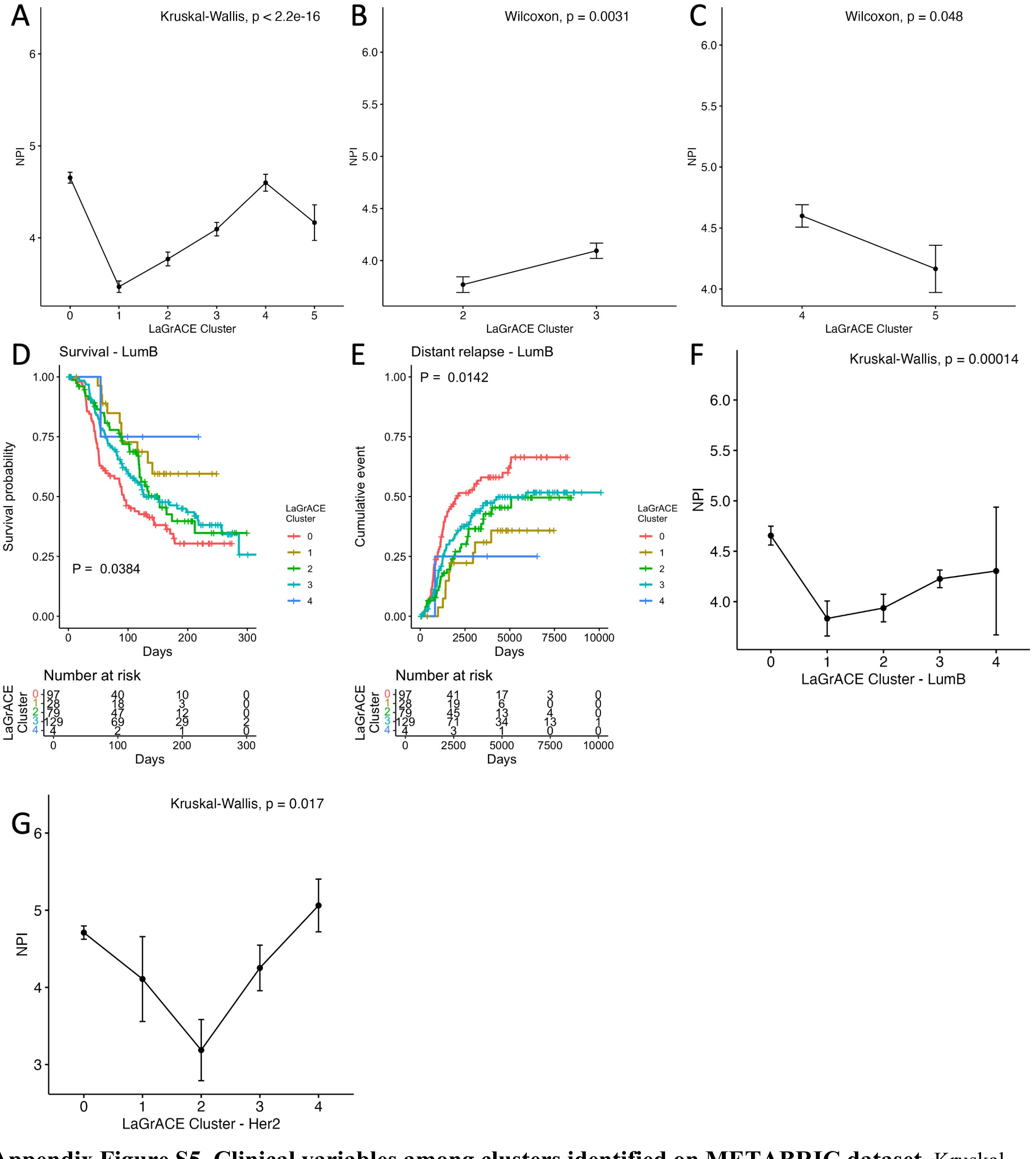

Appendix Figure S5. Clinical variables among clusters identified on METABRIC dataset. Kruskal-Wallis test or Wilcoxon rank-sum test was performed comparsion of NPI.

(A) Line plot of Nottingham Prognostic index (NPI) for all clusters (all samples).
(B) Line plot of NPI for cluster 2 and cluster 3 (all samples).
(C) Line plot of NPI for cluster 4 and cluster 5 (all samples).
(D) Survival analysis for Luminal B samples stratified by LaGrACE clusters.
(E) recurrent event analysis for Luminal B samples stratified by LaGrACE clusters.
(F) Line plot of Nottingham Prognostic index (NPI) for all clusters (Luminal B sample only).
(G) Line plot of Nottingham Prognostic index (NPI) for all clusters (HER2+ sample only).

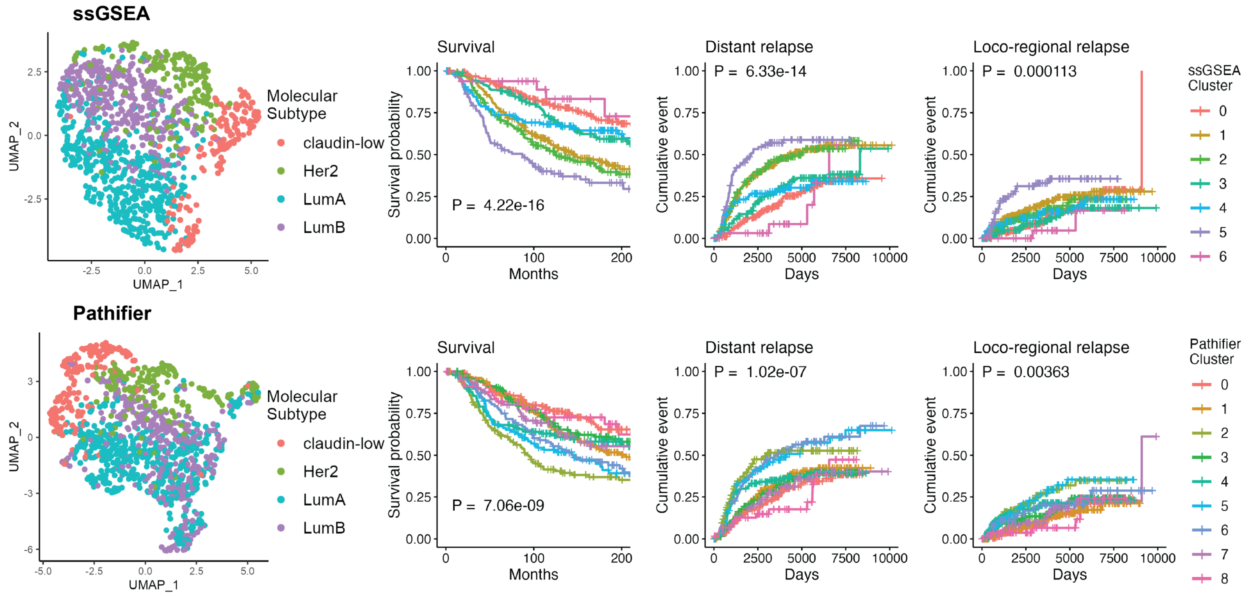

Appendix Figure S6. Separation of METABRIC breast cancer samples and Kaplan-Meier estimate of survival and recurrent event by ssGSEA's and Pathifier's clusters

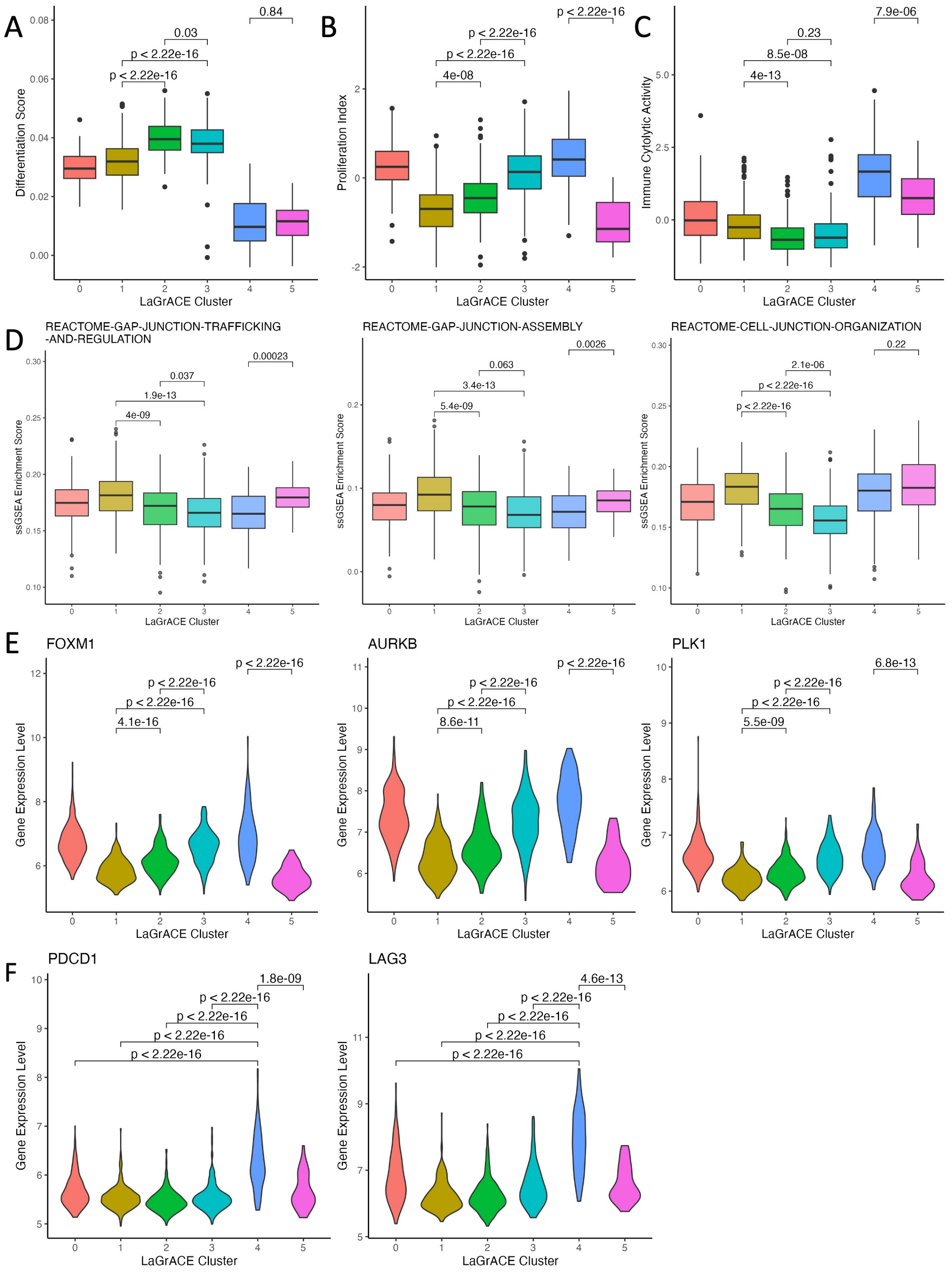

Appendix Figure S7. Biological process and molecular mechanism difference among identified clusters in breast cancer. Box plots of (A) differentiation, (B) proliferation and (c) immune cytolytic scores of each sample, (D) ssGSEA pathway enrichment score of junction-associated pathways; Violin plots visualizing gene expression of (E) FOXM1, AURKB and PLK1 and (F) immune exhaustion markers, PDCD1 and LAG3. Wilcoxon rank-sum test was conducted to calculate P values.

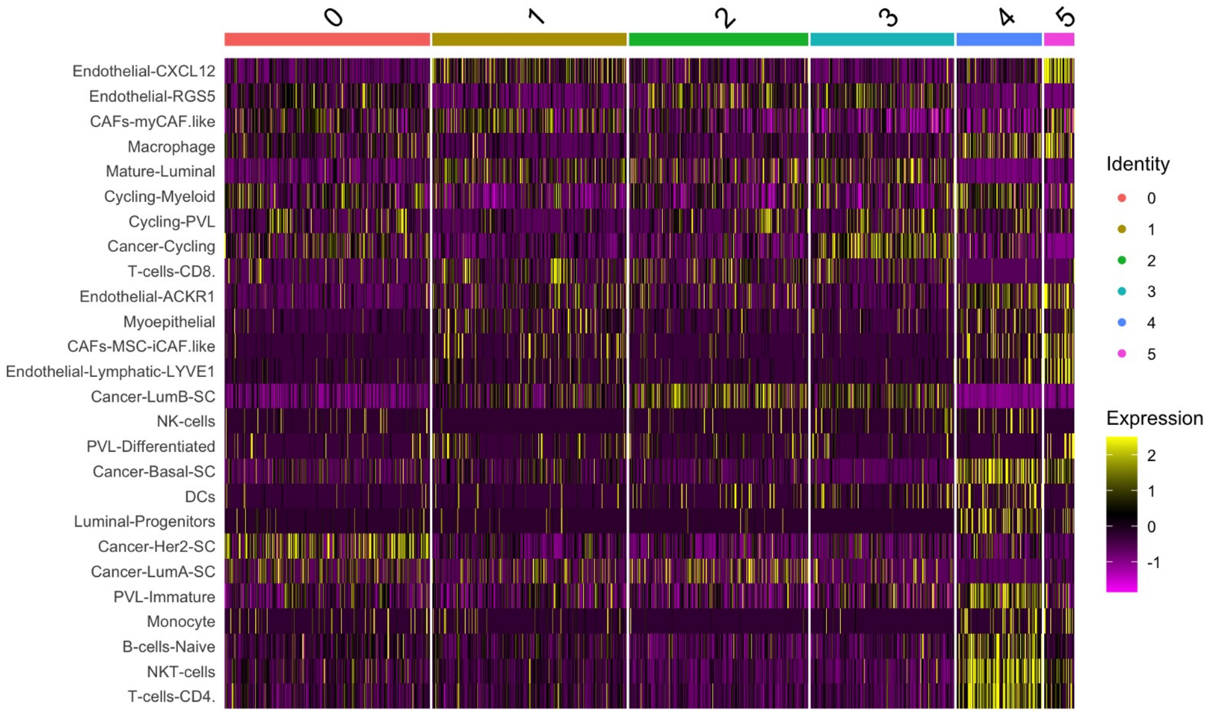

Appendix Figure S8. Heatmap of scaled predicted cell type fractions inferred using CIBERSORTx.

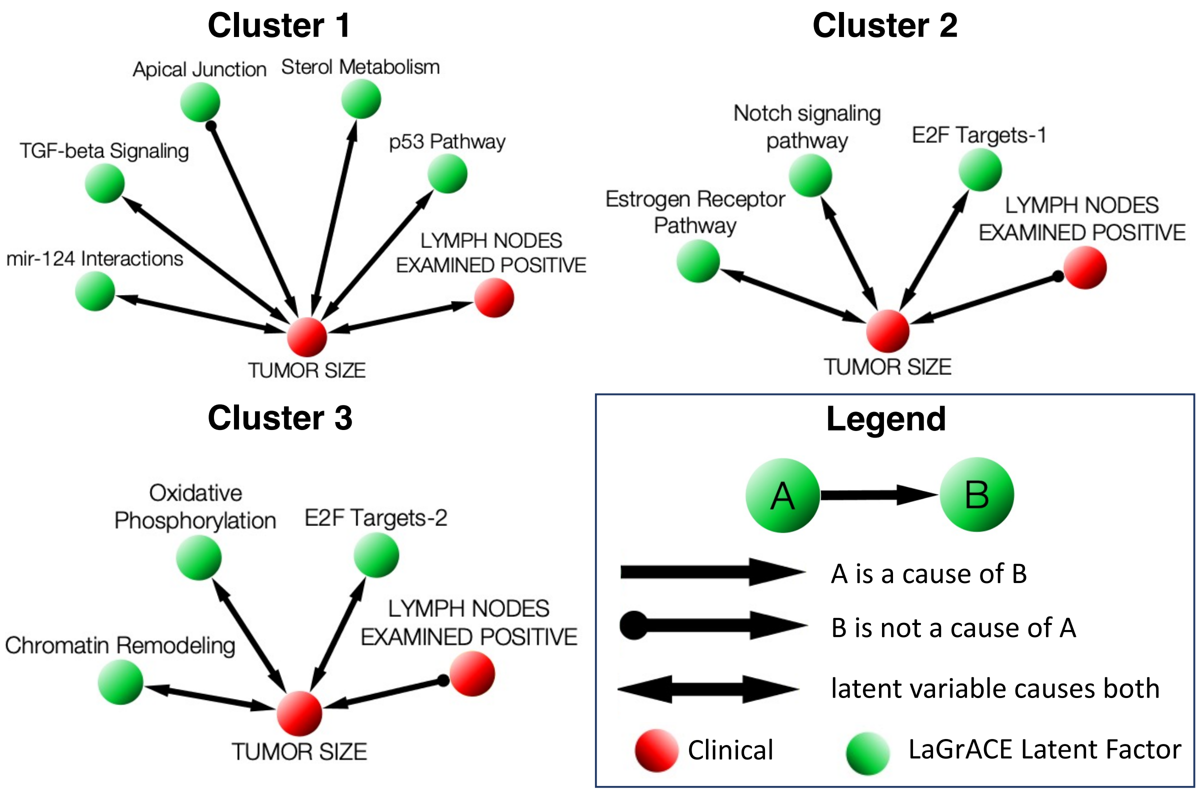

Appendix Figure S9. The cluster specific causal graphs for luminal clusters (1, 2 & 3). Note that besides the edges represented by a direct arrow (A→B), all other edges do not exclude the possibility of a latent confounder. AGE AT DIAGNOSIS: Age of patient at cancer diagnosis (years); LYMPH NODEs Examined Positive: Number of lymph nodes where tumor cells are seen: 0, 1 to 3, 4 to X; TUMOR SIZE: Log2 size of tumor (cm)

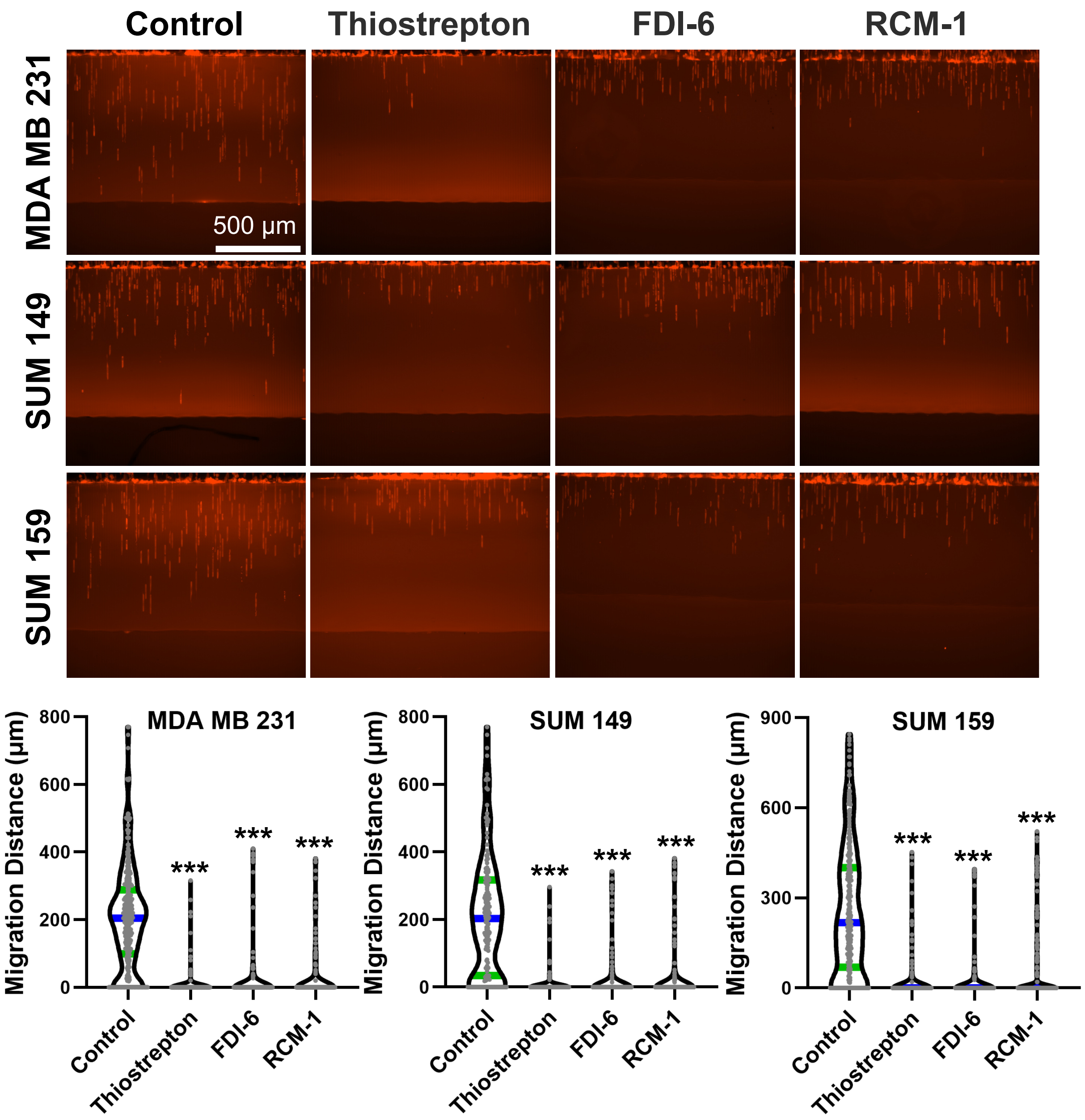

Appendix Figure S10. FOXM1 inhibitors inhibit the cell migration of breast cancer cells. (Scale bar: 500 µm) Each dot represents the cell migration distance in a channel. The green bar represents the median, and the blue bars represent the quartiles. (n = 200 channels). *** refers to $P < 0.001$ (compared with control, non-parametric Mann-Whitney U test).

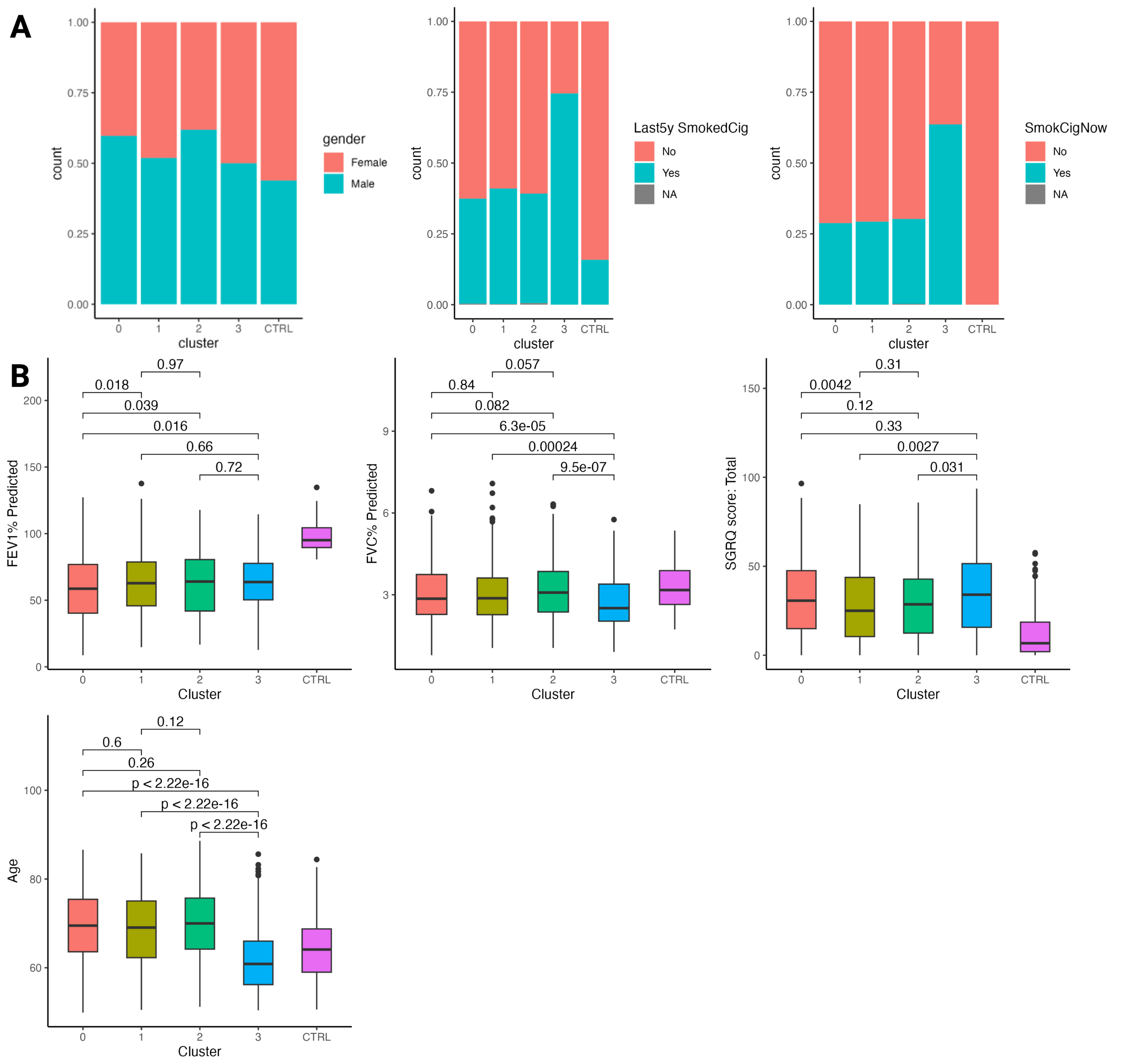

Appendix Figure S11. Association between LaGrACE cluster and clinical variables. (A) Box plots illustrating categorical variables: Gender, Current Smoking Status, and Smoking Status over the Past 5 Years. (B) Box plots depicting continuous variables including Predicted FEV1, Predicted FVC, SGRQ Score, and age. Wilcoxon rank-sum test was conducted to calculate P values.

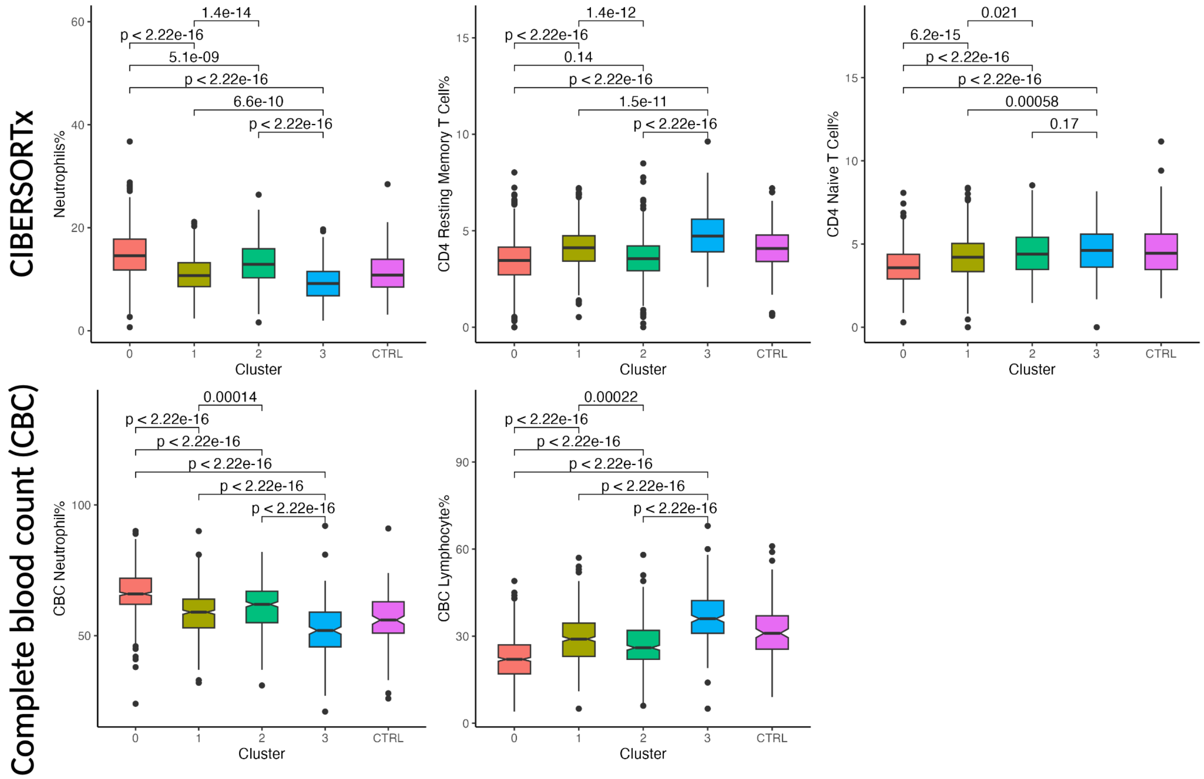

Appendix Figure S12. Association between LaGrACE cluster and white blood cell type fraction. Wilcoxon rank-sum test was conducted to calculate P values.

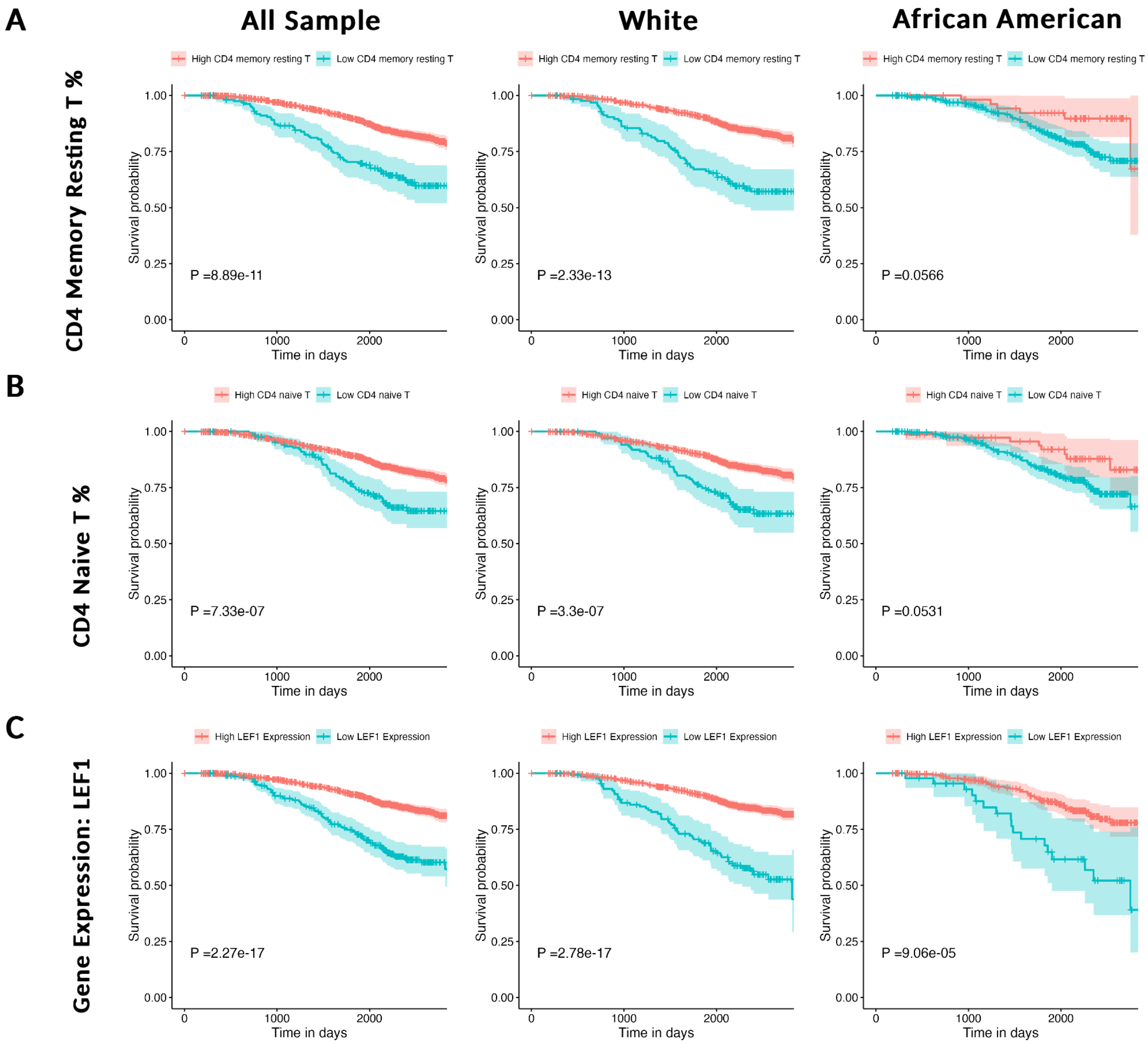

Appendix Figure S13: Kaplan-Meier survival curve analysis for COPD patients. This figure presents the estimation of survival curves based on three different criteria: (A) the fraction of CD4+ memory resting T cells, (B) the fraction of CD4+ naïve T cells, and (C) the expression levels of the LEF1 gene. An optimal cutpoint was determined for each combination of feature and race.

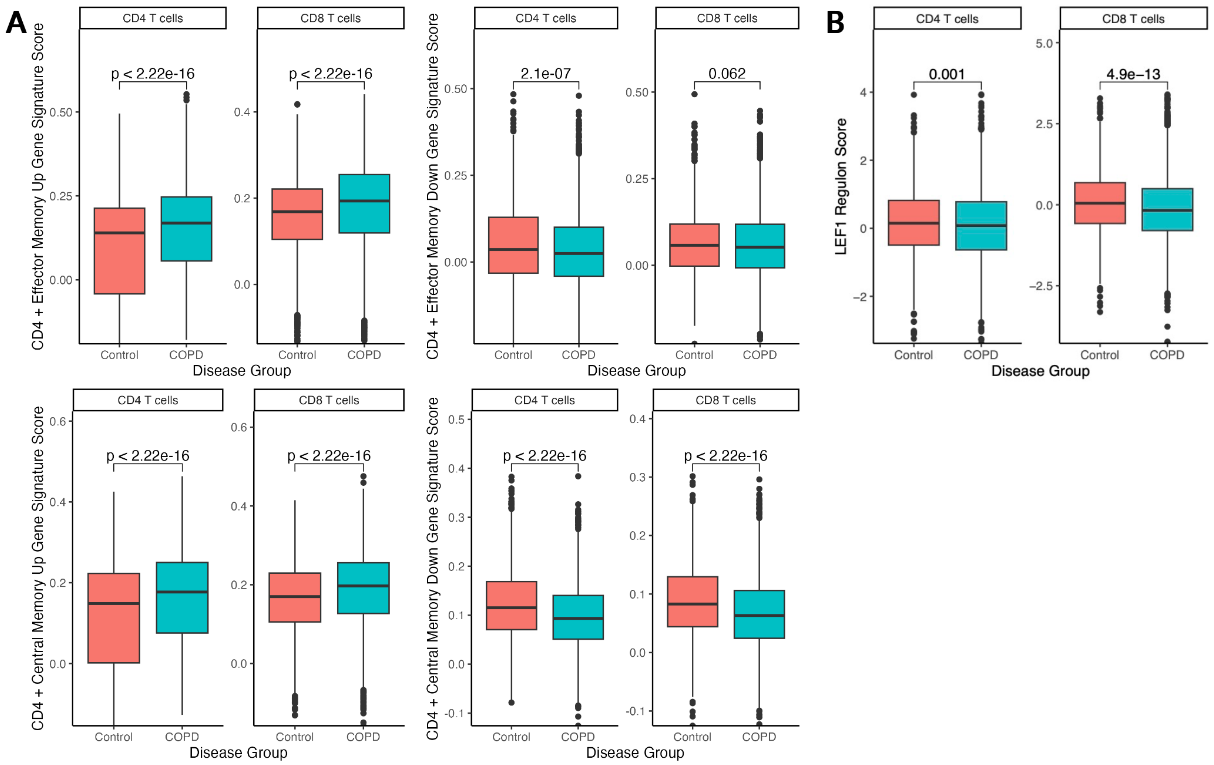

Appendix Figure S14: COPD module score and LEF1 regulon score in CD4 and CD8 T cells from human lung tissue. (A) The COPD module score, calculated based on COPD associated differentially expressed genes identified in memory CD4 T cells from blood. (B) The LEF1 regulon score. For an accurate assessment, the analysis is restricted to samples from individuals aged 55 to 70 years (9 control and 17 COPD samples). *p*-values: Wilcoxon rank-sum test.
